# Surgical exploration and discovery program: inaugural involvement of otolaryngology – head and neck surgery

**DOI:** 10.1186/s40463-015-0059-5

**Published:** 2015-02-03

**Authors:** Brittany Greene, Linden Head, Nada Gawad, Stanley J Hamstra, Laurie McLean

**Affiliations:** University of Ottawa Faculty of Medicine, 451 Smyth Road, Ottawa, Canada; University of Toronto Department of Surgery, Faculty of Medicine, 1 King’s College Circle, Medical Sciences Building, Room 2109, Toronto, Canada; Department of Surgery, The Ottawa Hospital, General Campus, 501 Smyth Road, Ottawa, Canada; University of Ottawa Skills and Simulation Centre, The Ottawa Hospital, Civic Campus, Loeb Research Building, 1st floor, 725 Parkdale Avenue, Ottawa, Canada; Department of Otolaryngology Head and Neck Surgery, The Ottawa Hospital, General Campus, 501 Smyth Road, Ottawa, Canada

**Keywords:** Undergraduate medical education, Surgical exploration and discovery, Simulation

## Abstract

**Background:**

There is significant variability in undergraduate Otolaryngology – Head and Neck Surgery (OTOHNS) curricula across Canadian medical schools. As part of an extracurricular program delivered jointly with other surgical specialties, the Surgical Exploration and Discovery (SEAD) program presents an opportunity for medical students to experience OTOHNS. The purpose of this study is to review the participation and outcome of OTOHNS in the SEAD program.

**Methods:**

The SEAD program is a two-week, 80-hour, structured curriculum that exposes first-year medical students to nine surgical specialties across three domains: (1) operating room observerships, (2) career discussions with surgeons, and (3) simulation workshops. During observerships students watched or assisted in surgical cases over a 4-hour period. The one-hour career discussion provided a specialty overview and time for students’ questions. The simulation included four stations, each run by a surgeon or resident; students rotated in small groups to each station: epistaxis, peritonsillar abscess, tracheostomy, and ear examination. Participants completed questionnaires before and after the program to evaluate changes in career interests; self-assessment of knowledge and skills was also completed following each simulation. Baseline and final evaluations were compared using the Wilcoxon Signed-Rank test.

**Results:**

SEAD participants showed significant improvement in knowledge and confidence in surgical skills specific to OTOHNS. The greatest knowledge gain was in ear examination, and greatest gain in confidence was in draining peritonsillar abscesses. The OTOHNS session received a mean rating of 4.8 on a 5-point scale and was the most popular surgical specialty participating in the program. Eight of the 18 participants were interested in OTOHNS as a career at baseline; over the course of the program, two students gained interest and two lost interest in OTOHNS as a potential career path, demonstrating the potential for helping students refine their career choice.

**Conclusions:**

Participants were able to develop OTOHNS knowledge and surgical skills as well as refine their perspective on OTOHNS as a potential career option. These findings demonstrate the potential benefits of OTOHNS departments/divisions implementing observerships, simulations, and career information sessions in pre-clerkship medical education, either in the context of SEAD or as an independent initiative.

**Electronic supplementary material:**

The online version of this article (doi:10.1186/s40463-015-0059-5) contains supplementary material, which is available to authorized users.

## Background

The pre-clerkship Otolaryngology – Head and Neck Surgery (OTOHNS) curriculum in Canadian undergraduate medical education has been examined recently by the Canadian Society of OTOHNS (CSO) Undergraduate Medical Education (UME) Working Group [1]. The authors found significant variation exists between schools across the country [1]. At the pre-clerkship level, hours dedicated to OTOHNS teaching ranges from 0 to 50, and there is substantial variability in format of teaching delivered [1]. As many as 7 Canadian medical schools provide 10 or fewer hours of formal OTOHNS teaching [1]. Studies have shown that poor early exposure and minimal involvement of surgeons in pre-clerkship education are barriers to creating interest in the field [2-4]. Also, importantly, the majority of medical students predict their specialty choice prior to clerkship [5].

The Surgical Exploration and Discovery (SEAD) program provides more experiential learning opportunities for pre-clerkship medical students interested in surgical careers, through operating room (OR) observerships, career discussions, and simulation-based workshops [6]. The program was founded at the University of Toronto in 2012 and has run successfully there for three years [6]. In June of 2014, the University of Ottawa Skills and Simulation Centre (uOSSC) collaboratively with the University of Ottawa Faculty of Medicine, Department of Otolaryngology – Head and Neck Surgery and Department of Surgery, initiated the first Canadian expansion of the SEAD program. At the University of Ottawa, the program maintained the overall structure of the program as implemented in Toronto, with some variation in specialties included and the workshop content.

OTOHNS is a unique surgical specialty in Canada in that at some institutions it is its own department, while at others it is a division within the Department of Surgery. As such, its inclusion into surgical education programs can be variable. In past SEAD programs at other institutions, OTOHNS was not included in the curriculum. However, as a direct-entry surgical specialty, the inclusion of OTOHNS in the SEAD program is vital to the underlying objective of SEAD: to facilitate informed career decision-making for students interested in surgery. Thus, through the collaborative efforts of both the Department of OTOHNS and the Department of Surgery, OTOHNS was included for the first time in any SEAD program in 2014 at the University of Ottawa.

The purpose of this study is to review the participation and outcome of OTOHNS in the University of Ottawa SEAD program. The findings of this study may help to inform OTOHNS departments and divisions considering implementing simulation-based learning and career information sessions in UME, either in the context of SEAD or as an independent initiative.

## Methods

### SEAD program curriculum at the University of Ottawa

The SEAD Program is a two-week summer program for students who have completed their first year of medical school. All divisions within the Department of Surgery (General Surgery, Plastic Surgery, Orthopedic Surgery, Urology, Neurosurgery, Cardiac Surgery, Vascular Surgery, Thoracic Surgery) as well as Otolaryngology – Head and Neck Surgery were included. Over the course of the two weeks, students were exposed to nine surgical specialties across three domains:

1. *Operating room observerships:* students spent one morning (8 am – 12 pm) observing each of the specialties in the OR.
2. *Career discussions:* over lunch (12 pm – 1 pm) a surgeon provided a career discussion and answered questions on their respective specialty. The discussion covered training, fellowships, scope of practice, daily responsibilities, and work-life balance. Each specialty provided one session.
3. *Simulation workshop:* each afternoon (1 pm – 4 pm) the specialty providing the career discussion would proceed to run a hands-on, simulation workshop. The goals of the sessions were to provide exposure to common procedures, develop the students’ skills, and stimulate interest in the specialty.

At the end of the two week program all students had completed an observership, and participated in a career discussion and simulation for each of the nine participating surgical specialties; the detailed schedule for the program can be found in Additional file 1.

All program participants were given an informational manual (68 pages) at the outset of the program, which provided an overview of the information covered in each of the nine specialties’ career discussion and simulation workshop. The OTOHNS segment was 5 pages. It included a description of the specialty, residency and fellowship training programs, brief descriptions of common procedures and an outline of the stations at the simulation workshop. Students were also provided with workshop objectives to guide preparation, and a list of reference books available online to review surgical anatomy prior to their OR observership.

### OTOHNS SEAD curriculum

#### Career discussion

Two otolaryngologists, one with an academic practice and the other primarily community-based, facilitated the lunchtime career discussion. The setting was informal. Participants were encouraged to ask questions.

#### Simulation workshop

The 3-hour simulation workshop involved four stations. Participants rotated through stations in groups of 4–5, every 45 minutes. Facilitators remained at one station for the duration of the workshop.

##### Epistaxis Station (two resident facilitators)

At this station, there were two plastic head models. Bleeding was simulated through an IV attached inside the nose. Students were provided with a nasal packing tray. Residents provided teaching as per the objectives in Table 1.

**Table 1:** **Goals of Epistaxis station**

**Knowledge**	**Skills**	**Attitudes**
List the blood supply to the nose.	List/identify the instruments/medications required to perform nasal packing and set up a tray accordingly.	Epistaxis is common and will be encountered by most physicians regardless of specialty.
Identify Kiesselbach’s plexus/Little’s area.		Can be life threatening, Recognize importance of identifying bleeding source and doing a good pack.
	Learn how to hold a nasal speculum, bayonet forceps, nasal suction.	
		Recognize what constitutes a poor pack.
Recognize the difference between anterior and posterior epistaxis.	Learn how to examine the nose (anterior rhinoscopy).	
	Learn how to place local anesthetic/vasoconstrictor in the nose.	
	Compare and contrast various nasal packs.	
List the risk factors for epistaxis.	Learn how to place an anterior pack.	
	Review complications of nasal packing.	
List and explain the treatment options for acute management of anterior epistaxis.		
List and explain the treatment options for acute management of posterior epistaxis.		
Describe how to potentially prevent epistaxis.		

##### Peritonsillar Abscess Station (one resident facilitator)

A low fidelity model that was built in-house was used to demonstrate a peritonsillar abscess [7]. A balloon filled with lotion was set behind a latex mold resembling the oropharynx which was then inset within a box to mimic the approach through the oral cavity. Students were provided with a procedure tray. The resident provided teaching as per the objectives in Table 2.

**Table 2:** **Goals of Peritonsillar Abscess (PTA) station**

**Knowledge**	**Skills**	**Attitudes**
Recognize how infectious tonsillitis may affect other organ systems.	List/identify the instruments/medications required to drain a PTA and set up a tray accordingly.	Peritonsillar abscess drainage is a straightforward procedure that Family Medicine, Emergency, and OTOHNS should be able to perform.
		Many communities do not have OTOHNS MDs, so the more MDs that can successfully do this procedure, the better the patient care.
	Identify a PTA.	
	Identify the most likely location of a PTA and the landmarks for your aspiration/incision and drainage.	
		Understand peritonsillar anatomy so that fear of performing the procedure is decreased
Explain how to grade the size of tonsils.		
	Topically anesthetise the oropharynx.	
Compare and contrast the clinical presentation, diagnosis, and treatment of tonsilloliths, peritonsillar cellulitis, PTA, and mononucleosis.	Inject local anesthetic into the soft palate.	
	Incise and drain a PTA.	
	Review complications of PTA drainage.	

##### Tracheostomy Station (one staff surgeon facilitator)

Cadaveric porcine tracheas were used as models and students were provided with a tracheostomy tray and cuffed tracheostomy tube. Students were paired such that one acted as the primary surgeon and the other as assistant. Students completed the procedure then switched roles. The surgeon provided teaching as per the objectives in Table 3.

**Table 3:** **Goals of Peritonsillar Abscess (PTA) station**

**Knowledge**	**Skills**	**Attitudes**
Identify the parts of a tracheostomy tube including: inner cannula, introducer, tracheostomy tube, phalanges, tracheostomy tie, cuff, and cork.	List/identify the instruments/medications required to perform a tracheostomy/cricothyroidotomy and set up a tray accordingly.	Airway obstruction is life threatening.
		Be safe and calm under pressure.
		Important to work as a team.
		Consider multidisciplinary care (respiratory therapy, nursing, anaesthesia).
	Identify the landmarks for a tracheostomy/cricothyroidotomy.	
	Carry out a stepwise approach to a tracheostomy/cricothyroidotomy.	
	Learn how to safely change a tracheostomy tube.	
Compare and contrast tracheostomy and cricothyroidotomy.		
	Review complications of tracheostomy/cricothyroidotomy (acute and chronic).	
List three indications for placement of a tracheostomy tube.		

##### Ear Exam Station (one staff surgeon facilitator)

The OtoSim™ and a diagram of the temporal bone in cross section were used at this station. Participants had no prior knowledge of ear and its exam. The surgeon provided teaching as per the objectives in Table 4.

**Table 4:** **Goals of Peritonsillar Abscess (PTA) station**

**Knowledge**	**Skills**	**Attitudes**
Identify structures of the external and middle ear.	Properly use an otoscope.	Gain proficiency in basic otoscopy.
	Perform an otologic exam.	
	Identify landmarks of the external and middle ear (normal and diseased).	

### Participants

SEAD Program participants consisted of 18 students who had just completed their first year of medical school at the University of Ottawa. Participants were selected based on a written application outlining their desire to participate in a surgical education program; there were 29 students who applied for 18 spots. The number of positions available was determined based on the capacity of each specialty to accommodate students in the ORs.

Participation in the program was voluntary. Institutional ethics approval was received, and written informed consent was obtained from all participants.

### Evaluations

Evaluation OTOHNS’s involvement in the SEAD program consisted of three components:

1. Entry questionnaire regarding baseline demographics, surgical experience (e.g. observerships, undergraduate education), and specialties of interest;
2. Two OTOHNS specific evaluation forms
3. Exit questionnaire to determine the influence of the program on career interests

#### OTOHNS evaluation design

Two forms, evaluating participants’ reaction to the programming and their learning [8], were provided to students to complete the day after participating in the OTOHNS lunchtime career discussion and afternoon simulation workshop. The same two forms were used to evaluate each of the specialties included in SEAD the day after the specialty-specific career discussion and workshop.

1. A standardized evaluation form developed and used broadly by the uOSSC to measure participants’ reaction to any simulation session held there. The form is based on a 5-point Likert Scale. Students were asked to quantify reactions to overall quality (1 – poor; 5 – excellent), and specific elements of the day such as time, equipment, objectives (1 – strongly disagree; 5 – strongly agree). There was also a space for written comments. The detailed form can be found in Additional file 2.
2. A second evaluation form was created to evaluate self-reported learning of knowledge and skills as well as reaction. Students were asked to rate their knowledge and confidence (before and after the activity) of different topics in OTOHNS related to each simulation on a 10-point scale (none to very high). Students were asked to describe their reaction to elements of the day on a 5-point Likert Scale (Strongly Disagree to Strongly Agree). The detailed form can be found in Additional file 3.

#### Career interest questionnaire design

As part of the entry questionnaire, students were asked to indicate which surgical specialties they were interested in pursuing as a career. The 9 surgical specialties of the program were listed as options. Students could select as many as they wanted. The identical question was repeated on the exit questionnaire.

### Statistical analysis

The Wilcoxon Signed-Rank test was used to compare non-parametric paired data for differences in baseline and final test results. A p value of < 0.05 was indicative of statistical significance. All statistical evaluation was performed with SPSS software.

## Results

Eighteen first-year medical students completed the SEAD Program. Baseline demographics and surgical experience of the participants are outlined in Table 5.

**Table 5:** **Baseline demographics and surgical experience of SEAD participants (n = 18)**

Gender	Male	7
	Female	11
Age	20 to 22	12
	23 to 25	2
	>25	4
Education	Bachelor’s degree	14
	Master’s degree	3
	PhD	1
Cases observed prior to participating in SEAD program	0	0
	1 to 5	5
	6 to 10	6
	11 o 15	1
	16 to 20	5
	>20	1
Number of surgical specialties observed prior to participating in SEAD program	Mean	2.22
	Median	2
	Range	4
Interest in a surgical career prior to participating in SEAD program	Very interested	13
	Somewhat interested	5
	Not interested	0
Learned suturing skills prior to participating in SEAD program	No	4
	Yes, before medical school	2
	Yes, suturing workshops in medical school	12
Participated in Simulation Session(s) prior to participation in SEAD program	No	16
	Yes	2

Students’ self-reported change in knowledge over the course of the program is reported in Table 6. There was a significant difference between the pre- and post-activity measures on all knowledge dimensions. The overall mean difference in knowledge pre and post was 3.0, with knowledge in ear examination experiencing the largest change at 5.2; the smallest change was observed in knowledge of development needs.

**Table 6:** **Self-reported knowledge of key concepts**

	**Before activity, mean (SE)**	**After activity, mean (SE)**	**Difference mean (SE)**	**p-value**
1. Knowledge of Otolaryngology - Head and Neck Surgery as a Career	4.7 (0.4)	7.8 (0.2)	3.1 (0.4)	<0.001
2. Knowledge of Epistaxis	5.9 (0.4)	8.3 (0.2)	2.4 (0.4)	<0.001
3. Knowledge of Airway Obstruction and Tracheostomy	5.1 (0.4)	8.0 (0.3)	2.9 (0.3)	<0.001
4. Knowledge of Examining the Ear	2.1 (0.3)	7.3 (0.4)	5.2 (0.4)	<0.001
5. Knowledge of Tonsillitis, Peritonsillar Cellulitis and Peritonsillar Abscess	5.2 (0.4)	8.3 (0.3)	3.1 (0.4)	<0.001
6. Knowledge of your own strengths and development needs	5.6 (0.5)	7.3 (0.3)	1.7 (0.5)	0.003
**Mean**	**4.8 (0.2)**	**7.8 (0.1)**	**3.0 (0.2)**	<0.001

Students’ self-reported change in confidence in clinical skills over the course of the program is reported in Table 7. There was a significant difference between the pre- and post-activity measures on all confidence dimensions. The overall mean difference in confidence pre and post was 3.9, with confidence in draining peritonsillar abscess experiencing the largest change at 4.9; the smallest change was observed in overall confidence in surgical skills.

**Table 7:** **Self-reported confidence in clinical skills**

	**Before activity, mean (SE)**	**After activity, mean (SE)**	**Difference mean (SE)**	**p-value**
1. Confidence in your ability to manage epistaxis	3.2 (0.4)	7.2 (0.3)	3.9 (0.4)	<0.001
2. Confidence in your ability to perform a tracheostomy	2.0 (0.3)	5.8 (0.4)	3.8 (0.3)	<0.001
3. Confidence in your ability to examine the ear	2.4 (0.5)	7.1 (0.5)	4.7 (0.5)	<0.001
4. Confidence in your ability to drain a peritonsillar abscess	2.0 (0.3)	6.9 (0.4)	4.9 (0.4)	<0.001
5. Confidence in overall surgical skills	4.3 (0.5)	6.5 (0.3)	2.2 (0.5)	0.001
**Mean**	**2.8 (0.2)**	**6.7 (0.2)**	**3.9 (0.2)**	<0.001

**Figure 1:**
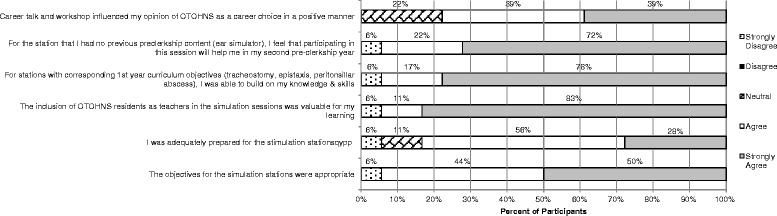
**Student feedback of the simulation session and career talk**

Student feedback about the simulation session and career talk is reported in Table 8 and Figure 1. Students rated the session very positively, with an overall mean of 4.8 (5-point scale). The OTOHNS session was the highest rated session over the course of the two-week program; session ratings ranged from 4.0 to 4.8.

**Table 8:** **Student feedback of simulation session**

**Domain**	**Mean (SE)**
Overall quality	4.7 (0.1)
Clear & informative lecture/demo	4.7 (0.1)
Clear objectives	4.9 (0.1)
Objectives met	4.8 (0.1)
Instructor knowledgeable & informed	5.0 (0.0)
Time	4.6 (0.2)
Feedback	4.8 (0.1)
Teaching ratio	4.9 (0.1)
Equipment	4.7 (0.2)
**Mean**	**4.8 (0.04)**

The median net change in interest was −1 overall, for all specialties (Table 9). At baseline and following the program, OTOHNS had the largest number of interested students (10). Over the course of the program two students lost interest in OTOHNS as a potential career path and two new students gained interest, for an overall net change of 0.

**Table 9:** **Interest by surgical specialty before and after SEAD**

**Surgical specialty**	**No. interested at baseline**	**Final no. Interested**	**No. that developed a new interest**	**No. that ruled out a prior interest**	**Net change in no. interested**
OTOHNS	10	10	2	2	0
Plastic surgery	7	8	1	0	+1
Orthopaedic surgery	9	7	1	3	−2
Cardiac surgery	6	2	0	4	−4
Vascular surgery	7	9	5	3	+2
Neurosurgery	2	4	3	1	+2
Thoracic surgery	7	2	2	7	−5
General surgery	10	9	2	3	−1
Urology	5	3	1	3	−2
**Median**	**7**	**7**	**2**	**3**	**−1**

## Discussion

Inclusion of OTOHNS in the SEAD program is novel to the University of Ottawa. While the SEAD program is relatively new itself, it presents a unique opportunity to provide meaningful exposure to the surgical specialties for medical students early in their training. Given that there is substantial variability in OTOHNS undergraduate medical education at the pre-clerkship level, including OTOHNS in the SEAD program creates an interesting platform to enhance students’ learning and promote it as a career choice.

### Building OTOHNS knowledge and confidence

OTOHNS learning in the SEAD program is significant and valuable to participants. According to self-reported rating of knowledge in the four simulation stations, participants report a significant improvement. Students also report a significant improvement in their confidence to perform the skills learned in each of the stations. As expected, at baseline, students rated their knowledge higher than their confidence to perform the corresponding skill. A greater magnitude of growth in confidence was noted than in knowledge. Significant gains in skill-related confidence were observed after just 45 minutes of simulation for each skill.

The largest gain in knowledge was documented for the ear exam station. Ear anatomy and pathology is taught primarily in the second year of the pre-clerkship curriculum. The other 3 stations had corresponding 60–90 minute lectures on the content in the first year of the pre-clerkship curriculum. Consequently, the OtoSim™ was many students’ first exposure to the anatomy of the ear. Access to simulation-based learning for pre-clerkship medical students is highly limited. In the future, it may be worthwhile integrate simulation-based learning into the core curriculum.

The largest gain in confidence was in draining a PTA. While learning of attitudes was not directly measured, the large gain in confidence may act as a surrogate measure. Students seem to have learned the attitude that PTA drainage is a straightforward procedure that does not always require a specialist. After the workshop, participants rated their confidence in ability to perform a tracheostomy lowest, compared to the other stations. This suggests students have some insight into their skills as well as risks and complications of procedures.

### OTOHNS career interest

Among SEAD students there was a very strong interest in OTOHNS as a potential career path; at the outset and close of the program, OTOHNS had the highest number of interested students of all surgical specialties. It is important to note that the level of interest among SEAD participants is likely not representative of level of interest among the entire first-year class. The competitive application process for a limited number of spots in this extracurricular program creates a selection bias for students who have already identified an interest in surgical specialties. The source of this interest among surgically inclined students could be a result of the relatively strong presence of OTOHNS in the University of Ottawa UME pre-clerkship curriculum. In the pre-clerkship curriculum there are 50 hours of OTOHNS instruction, which is the highest amount of OTOHNS pre-clerkship instruction offered at any Canadian medical school [9].

Despite high interest levels in pre-clerkship, historically, there has been an average of two students from the University of Ottawa to apply to OTOHNS as a first choice residency program [10]. This is the first time that there is an indication of the number of first year students interested in OTOHNS. Although there are limitations in comparing these two groups of students, as SEAD participants could select several specialties on the questionnaire, the observation suggests that there is attrition of candidates from the University of Ottawa considering OTOHNS as a career choice as they progress through their undergraduate education. There could be several factors contributing to this phenomenon. First, OTOHNS is highly represented in pre-clerkship at the University of Ottawa, with significant lecture and workshop time. Conversely, students’ main exposure to many of the other surgical specialties, such as plastic surgery and vascular surgery, occurs in clerkship. Thus, the high initial rate of interest may simply be because of early exposure to OTOHNS. Secondly, OTOHNS is a highly competitive specialty with only 30 residency positions available across Canada [10]. It is possible that some students self-select out of vying for an OTOHNS residency position. Third, it can be expected that as students explore other surgical and medical specialties, they modify their career interests as they make a more informed decision. While it has been documented that most students change their career choice between matriculation and graduation [11], it is unknown if other specialties see similar reductions in interest level throughout the undergraduate medical curriculum.

It is also important, however, to examine the role of clerkship in career decision-making as it has been demonstrated that positive clerkship experiences have a positive impact on preferences and attitudes towards a specialty as a career [12]. Compared to other surgical clerkship rotations (excluding Ophthalmology), where students are assigned to a discrete team (surgical clerk, junior resident, senior resident), the OTOHNS clerkship at the University of Ottawa is one week in duration. Students rotate through the different subspecialties of OTOHNS to obtain broad exposure. The amount of exposure to surgical aspects of OTOHNS is highly variable between individual students. These data provide an interesting opportunity for further investigation into the delicate balance of broad specialty exposure and the team participation. It also gives pause about opportunities for student exposure to OTOHNS in other Canadian programs that have no mandatory OTOHNS clerkship rotation.

Interesting to note, there was a median net loss in interest in surgery as a career choice by −1. Although some could criticize that the SEAD program resulted in one less student being interested in surgery, it should be viewed as a positive outcome in that one student has been provided the early opportunity to make a more informed career choice—a choice that has a better chance of resulting in a well-matched, well-suited physician who truly enjoys his/her career.

### Curriculum feedback and future opportunities

The participants’ reaction to OTOHNS as part of the SEAD program was very positive. Overall, the session was rated a mean of 4.8 on a 5-point scale and was the highest rated session over the course of the program. The OTOHNS workshop was most closely tied to the UME curriculum objectives; 94% of participants found the objectives appropriate. Appropriately tailored objectives may have set the students up for success, which led to high satisfaction with the session. It is also possible the rating could be inflated by its timing in the context of the whole SEAD program, as it was the first specialty-specific workshop (immediately following the two days of introductory skills).

When evaluating different elements of the program on a Likert scale, the most participants stated they ‘strongly agree’ that having residents as teachers in the simulation session was valuable for their learning. Residents can provide instruction that is appropriate for the learner’s level as they are of similar tenure. It also provides the added benefit of creating an opportunity for participants to ask more candid questions about the demands of the training program and lifestyle. For residents, leading stations at the workshop for SEAD participants creates a low-risk environment for them to develop their teaching skills.

Although the OTOHNS simulation was well received by students, evaluation of written and verbal feedback from various stakeholders (workshop facilitators, participants, simulation technicians) provides direction for opportunities to improve future sessions.

The PTA model was composed of a low-fidelity latex mold (designed to resemble the oropharynx) [7]. Creating a latex mold with representative anatomy proved challenging, hence it may be worthwhile to create a second-generation simulator based on lessons learned from this session (ie. use a simple printed image of the oropharynx in lieu of the mold). Given that the model is low tech and low cost, ideally the student:simulator ratio would approximate 1:1, ensuring that all students have ample opportunity to develop their skills. Overall, the model was an excellent tool for simulating a PTA and could potentially be used as a simulation for Emergency Medicine physicians as PTA is a common presentation to the Emergency Department.

Several students identified the tracheostomy station as the most exciting part of the simulation. In future sessions, it will be important to ensure that there are sufficient models to allow students to both perform and assist in the simulation. Also, it would be helpful to have examples of the numerous tracheostomy models that exist as well as the cricothyroidotomy kit that is found in the ED; this would serve to familiarize students with elements that they may encounter in their clerkship training. Similarly, for the epistaxis simulation it would be beneficial to orient students to headlights, Rapid Rhino, Nasal Epistaxis Double Balloon Catheter, and Floseal.

The OtoSim™ was well received by students, however given that they had no prior knowledge of ear anatomy and pathology, a brief online learning module completed the evening before might allow students to develop an understanding of basic anatomy in advance, allowing OtoSim™ to be an opportunity to apply their knowledge clinically.

Two stations that were not included in the simulation curriculum that would be interesting to explore the feasibility of for future sessions are a flexible fiberoptic laryngoscopy and a foreign body airway.

In the future, it would be valuable to formally evaluate the program feasibility from the preceptors’ perspective. However, from informal discussions with staff and residents, the additional burden of work was well distributed. The program is a student-led initiative. Each specialty was assigned a second-year medical student liaison that worked with the lead staff surgeon to organize their respective specialty’s participation. Two weeks of OR observerships had minimal impact on the surgeons’ daily schedule. Student leaders arranged the logistics of observership scheduling. Simulations were designed in collaboration with the simulation centre staff, surgeons, and student liaisons. Student liaisons developed the instructional manual. While the entire program was two weeks long, each speciality was only required to lead one afternoon, a 4-hour time commitment from 1–2 staff surgeons per specialty, to provide a career discussion and a workshop for all program participants. The cost of administering the program was divided between student participation fees, an external grant, and department funding, such that it was affordable for all parties involved. The majority of the cost was attributable to simulation centre rental fees, which would vary between institutions. The SEAD program has run successfully for 3 years at the University of Toronto, and now at the University of Ottawa. As evidence builds to support program’s value, future iterations may explore modifications to the core structure such that more students can be included, without compromising the quality of the experience.

## Conclusions

The inclusion of OTOHNS in the SEAD program at the University of Ottawa seemed to have been a success for all stakeholders. Students had the opportunity to develop their OTOHNS specific knowledge and surgical skills as well as refine their perspective on potential career options. The Department of OTOHNS had the opportunity build upon its formal UME curriculum as well as introduce concepts and skills to engaged students. OTOHNS residents also were provided a unique opportunity for teaching while providing a “real-life” perspective for students about OTOHNS residency. With continued expansion of the SEAD program to other medical schools, inclusion of OTOHNS should be considered to provide interested students with well-rounded exposure to OTOHNS and other surgical disciplines. Even in the absence of the SEAD program, an isolated OTOHNS simulation session and career discussion could provide many similar benefits for students and the Department alike.

## Additional files

Additional file 1:
**SEAD program schedule.**
Additional file 2:
**Student evaluation form.**
Additional file 3:
**OTOHNS specific student evaluation form.**

## Abbreviations

OTOHNS: Otolaryngology – Head and Neck Surgery
CSO: Canadian Society of OTOHNS
UME: Undergraduate Medical Education
SEAD: Surgical Exploration and Discovery
OR: Operating room
uOSSC: University of Ottawa Skills and Simulation Centre
