# Supplementary material for: Surgical exploration and discovery program: inaugural involvement of otolaryngology – head and neck surgery
Source: J Otolaryngol Head Neck Surg. 2015 Feb 3;44(1):3. doi: 10.1186/s40463-015-0059-5 (PMC4340281; doi:10.1186/s40463-015-0059-5)
Supplement: Additional file 1: — SEAD program schedule. [file 40463_2015_59_MOESM1_ESM.pdf]

Additional file 1 – SEAD program schedule

	Day 1	Day 2	Day 3	Day 4	Day 5
7:30 – 8:00	**Welcome Address** Chair of Surgery				
8:00 – 8:45	**Detailed program description**	**OR Observership**	**OR Observership**	**OR Observership**	**OR Observership**
8:45 – 10:00	**Study Introduction, Consent, Tests**				
10:00 – 11:00	**Scrub Session**				
11:00 – 11:30	**Travel to uOSSC**				
11:30 – 13:00	**Introduction to the Simulation Centre**	**Vascular Surgery Talk**	**Otolaryngology – Head and Neck Surgery Talk**	**Plastic Surgery Talk**	**Orthopedic Surgery Talk**
13:00 – 16:00	**Suturing Workshop**	**Instrument ID & Handling Workshop**	**Otolaryngology – Head and Neck Surgery Simulation**	**Plastic Surgery Simulation**	**Orthopedic Surgery Simulation**

	Day 6	Day 7	Day 8	Day 9	Day 10
8:00 – 12:00	**OR Observership**	**OR Observership**	**OR Observership**	**OR Observership**	**OR Observership**
12:00 – 13:00	**Cardiac Surgery Talk**	**Neurosurgery Talk**	**Thoracic Surgery Talk**	**General Surgery Talk**	**Urology Talk**
13:00 – 16:00	**Cardiac and Vascular Surgery Simulation**	**Neurosurgery Simulation**	**Thoracic Surgery Simulation**	**General Surgery Simulation**	**Urology Simulation**
16:00 – 18:00					**Closing Ceremonies & Post Test**
