# Supplementary material for: Surgical exploration and discovery program: inaugural involvement of otolaryngology – head and neck surgery
Source: J Otolaryngol Head Neck Surg. 2015 Feb 3;44(1):3. doi: 10.1186/s40463-015-0059-5 (PMC4340281; doi:10.1186/s40463-015-0059-5)
Supplement: Additional file 2: — Student evaluation form. [file 40463_2015_59_MOESM2_ESM.pdf]

Additional file 2 – Student evaluation form

Topic:
Date:
Time:
Location:
Lead facilitator:
Assistant facilitators:

EVALUATION

	1<br>Poor	2	3	4	5<br>Excellent
Please rate the overall quality of this presentation					

Please rate the following on a scale of 1 to 5.

	1<br>Strongly disagree	2	3	4	5<br>Strongly agree
The instructor provided a clear and informative lecture / demonstration					
The objectives of the session were clear					
The objectives of the session were met					
The instructor was knowledgeable and informed					
I had sufficient time to practice my technical skills					
I received adequate and appropriate feedback from my instructor					
There was an appropriate ratio of instructors to students					
There was adequate and appropriate equipment and models					

COMMENTS:
