# Supplementary material for: Surgical exploration and discovery program: inaugural involvement of otolaryngology – head and neck surgery
Source: J Otolaryngol Head Neck Surg. 2015 Feb 3;44(1):3. doi: 10.1186/s40463-015-0059-5 (PMC4340281; doi:10.1186/s40463-015-0059-5)
Supplement: Additional file 3: — OTOHNS specific student evaluation form. [file 40463_2015_59_MOESM3_ESM.pdf]

Additional file 3 – OTOHNS specific student evaluation form

SEAD PROGRAM
OTOLARYNGOLOGY—HEAD AND NECK SURGERY COMPONENT
PRE-POST PROGRAM ASSESSMENT

Thank you for evaluating the Otolaryngology—Head and Neck Surgery component of the SEAD program. Your responses will be valuable in further development of the program and will remain confidential.

<u>KNOWLEDGE OF KEY CONCEPTS</u>

Both BEFORE and AFTER the session, please rate your knowledge in each of the following:

1. Knowledge of Otolaryngology—Head and Neck Surgery as a career

	None		Little			Moderate			Very High	
**Before Activity**	1	2	3	4	5	6	7	8	9	10
**After Activity**	1	2	3	4	5	6	7	8	9	10

2. Knowledge of Epistaxis

	None		Little			Moderate			Very High	
**Before Activity**	1	2	3	4	5	6	7	8	9	10
**After Activity**	1	2	3	4	5	6	7	8	9	10

3. Knowledge of Airway Obstruction and Tracheostomy

	None		Little			Moderate			Very High	
**Before Activity**	1	2	3	4	5	6	7	8	9	10
**After Activity**	1	2	3	4	5	6	7	8	9	10

4. Knowledge of Examining the Ear

	None		Little			Moderate			Very High	
**Before Activity**	1	2	3	4	5	6	7	8	9	10
**After Activity**	1	2	3	4	5	6	7	8	9	10

5. Knowledge of Tonsillitis, Peritonsillar Cellulitis and Peritonsillar Abscess

	None		Little			Moderate			Very High	
**Before Activity**	1	2	3	4	5	6	7	8	9	10
**After Activity**	1	2	3	4	5	6	7	8	9	10

6. Knowledge of your own strengths and development needs

	None		Little			Moderate			Very High	
**Before Activity**	1	2	3	4	5	6	7	8	9	10
**After Activity**	1	2	3	4	5	6	7	8	9	10

<u>CONFIDENCE IN SKILLS IN OTOLARYNGOLOGY—HEAD AND NECK SURGERY</u>

Both BEFORE and AFTER the session, please rate your confidence in each of the following:

1. Confidence in your ability to manage epistaxis

	None		Little			Moderate			Very High	
**Before Activity**	1	2	3	4	5	6	7	8	9	10
**After Activity**	1	2	3	4	5	6	7	8	9	10

2. Confidence in your ability to perform a tracheostomy

	None		Little			Moderate			Very High	
**Before Activity**	1	2	3	4	5	6	7	8	9	10
**After Activity**	1	2	3	4	5	6	7	8	9	10

3. Confidence in your ability to examine the ear

	None		Little			Moderate			Very High	
**Before Activity**	1	2	3	4	5	6	7	8	9	10
**After Activity**	1	2	3	4	5	6	7	8	9	10

4. Confidence in you ability to drain a peritonsillar abscess

	None		Little			Moderate			Very High	
**Before Activity**	1	2	3	4	5	6	7	8	9	10
**After Activity**	1	2	3	4	5	6	7	8	9	10

5. Confidence in your overall surgical skills

	None		Little			Moderate			Very High	
**Before Activity**	1	2	3	4	5	6	7	8	9	10
**After Activity**	1	2	3	4	5	6	7	8	9	10

GENERAL EVALUATION:

PLEASE RANK YOUR OPINION ON THE LIKERT SCALE BELOW.

	STRONGLY DISAGREE	DISAGREE	NEUTRAL	AGREE	STRONGLY AGREE
The objectives for the SIMULATION STATIONS were appropriate					
I was adequately prepared for the SIMULATION STATIONS					
The inclusion of OTOHNS residents as teachers in the Simulation Sessions was valuable for my learning.					
For those stations that had corresponding first year curriculum content/objectives (tracheostomy, epistaxis, peritonsillar abscess), I feel I was able to build on my knowledge and skills					
For the station that I had no previous precklerkship content (ear simulator), I feel that participating in this session will help me in my second precklerkship year					
The career talk and workshop influenced my opinion of OTOHNS as a career choice in a POSITIVE MANNER					

1. In your opinion, what was the best part of the SIMULATION SESSION?

2. In your opinion, what was the worst part of the SIMULATION?

3. What (if any) changes would you make to the SIMULATION SESSION in the future?

4. What (if any) changes would you make to the CAREER DISCUSSION in the future?

5. If the CAREER DISCUSSION influenced your opinion of OTOHNS, please describe how so.

COMMENTS:
